# Cross-sectional study of passive opiate smoking in relation to stroke and some of stroke attributable risk factors in women

**DOI:** 10.1038/s41598-022-20861-w

**Published:** 2022-09-30

**Authors:** Nazanin Jalali, Parvin Khalili, Saeed Bahrampour, Mohammad Mahmoudabadi, Ali Esmaeili Nadimi, Zahra Jalali

**Affiliations:** 1 Non-Communicable Diseases Research Center, Rafsanjan University of Medical Sciences, Rafsanjan, Iran; 2 Department of Neurology, School of Medicine, Rafsanjani University of Medical Sciences, Rafsanjan, Iran; 3 Social Determinants of Health Research Center, Rafsanjan University of Medical Sciences, Rafsanjan, Iran; 4 Department of Epidemiology, School of Public Health, Rafsanjan University of Medical Sciences, Rafsanjan, Iran; 5 Student Research Committee, Rafsanjan University of Medical Sciences, Rafsanjan, Iran; 6 Department of Cardiology, School of Medicine, Rafsanjani University of Medical Sciences, Rafsanjan, Iran; 7 Department of Clinical Biochemistry, School of Medicine, Rafsanjan University of Medical Sciences, Rafsanjan, Iran

**Keywords:** Endocrinology, Medical research, Neurology, Risk factors

## Abstract

Opiate use is related to neuropathological disorders, stroke and stroke attributable risk factors. However, secondary exposure to opiate in relation to the above-mentioned complications is studied only in animal models and remains to be evaluated in human populations. We tested whether passive exposure to opiate is associated with stroke and the known stroke predictive factors. We carried out a cross-sectional study of 1541 never smoker women who participated in the Rafsanjan Cohort Study (RCS) with their husbands (2015–2017 recruitment phase). RCS is one of the 19 geographic districts of the Prospective Epidemiological Research Studies in Iran (PERSIAN cohort study). Unadjusted and adjusted multiple logistic regression analyses were performed to evaluate the relationship between second-hand opiate exposure (husband opiate smoking after marriage) and the odds ratio of stroke and the following stroke risk factors and predictive parameters: overweight/obesity (BMI > 25), cholesterol (chol) > 200 mg/dl, fasting blood sugar (FBS) > 125 mg/dl, low density lipoprotein (LDL) > 100 mg/dl, triglyceride (TG) >  = 150 mg/dl, hypertension, diabetes, and chronic headache. We observed a significant increased adjusted odds ratio (OR) of stroke (OR = 3.43, 95% CI:1.33–8.82) and its risk factors LDL > 100 mg/dl (OR = 1.37, 95% CI:1.01–1.87) and FBS > 125 mg/dl (OR = 1.58, 95% CI:1.08–2.30) in women associated with husbands’ opiate smoking. This relationship was observed after adjusting for the confounding parameters including age, education years, and first-degree family history of the relevant diseases. The increased odds ratio for stroke and high LDL displayed a dose-sensitive trend with years of husband’s opiate smoking after marriage (respective p-trends: 0.02 & 0.01). We did not observe a significant association between passive opiate smoking and high TG, high Chol or the diseases diabetes, hypertension and chronic headache. However, 89% increased odds ratio of chronic headache was observed to be associated with passive opiate smoking for more than 10 years (OR = 1.89, 95% CI:1.02–3.50). We found an increased risk of stroke and high LDL and FBS in women associated with passive opiate smoking. Furthermore, a dose-sensitive connection was found between the risks of stroke, high LDL and chronic headache with the years of passive opiate exposure. Our results point to the necessity of the future analyses, which further assess whether passive opiate exposure could be considered as an independent risk factor for stroke and metabolic diseases.

## Introduction

Lifestyle and environmental exposures have been extensively studied in relation to the risk of developing non-communicative chronic diseases that constituted the main cause of annual death in the twenty-first century before COVID-19 pandemic. Opiate use is one of the lifestyle exposures that is linked to the diseases such as stroke and cerebrovascular diseases^1–6^, atherosclerosis, obesity, diabetes, dyslipidemia and hypertension^7–11^. Additionally, opiate is also known to result in structural and molecular alterations in the cells of different regions of the brain and consequently result in neuropathological conditions such as executive and cognitive impairment^12–15^ and headache^16,17^. Opiate is suggested to exert its adverse metabolic and neurologic effects by some underlying biological mechanisms such as reduction in total antioxidant capacity, and induction of reactive oxygen species (ROS)^18–21^.

Opiate consumption in Asia and the Middle East is associated with rather low social stigma compared to the western countries^22,23^. It is most commonly administered via smoking, which is the fastest way to exert its neurological effects^23^. Many studies have focused on the association of firsthand opiate consumption (direct effects) with stroke, atherosclerosis, metabolic diseases and neurological dysfunctions^1–11^. However, the second-hand exposure effects on the above-mentioned complications are not studied in human populations.

Assessing the effects of parental opiate inhaling started before or after childbirth, we recently found a higher odds ratio of obesity in male young-adults exposed to parental opiate inhaling (started after childbirth) in a dose-dependent manner. Therefore, our previous results point to the effects of secondary opiate smoking on the risk of obesity in young subjects (15–35 years)^24^. Additionally, regarding the second-hand exposure to opiate, one study performed in rat animal models found that opium smoke exposure results in an increase in blood pressure and low density lipoprotein-cholesterol (LDL-chol)^25^.

Here, we decided to assess whether the wives of men smoking opiate regularly (after marriage date) display a difference in the risks of stroke, development of obesity, diabetes, hypertension, hyperglycemia, dyslipidemia and chronic headache as some of the known risk factors and predictive parameters of stroke^26,27^.

Comprehensive data collection on the history of illicit drug use by participants, is one of the strengths of the Rafsanjan Cohort Study (RCS)^28^, as one of the 19 geographically district areas of the Prospective Epidemiological Research Studies in Iran (PERSIAN)^29^. This benefit of RCS helped us study the relationship between the second-hand opiate smoking and stroke or some of its associated risk factors.

## Materials and methods

### Subjects, study design and ethical considerations

In this cross-sectional study, a total of 3261 husband-wife dyads were identified (35–70 years old), representing 32.64% of the total number of participants enrolled in the adult Rafsanjan Cohort Study (RCS) (3261/9990)^28^. All participants in this study were registered after providing a valid identification document (2015–2017 recruitment phase). The husband-wife relationship and the other family relationships are all accurately recorded in RCS by assigning accurate Personal Code Identification (PCID) numbers to all family members. Women with any history of substance use and cigarette smoking were excluded from our analysis (119/3261). All individuals reporting more than one marriage or the couples with different residential addresses, and with incomplete questionnaires were excluded from our study. Additionally, women with husband’s smoking cigarettes after marriage or consuming opiate with any administration route other than smoking were excluded from our analysis.

All procedures of invitation, interview, measurements and physical examinations were conducted according to the protocols of the PERSIAN cohort^29^, and under the supervision of the Iranian Ministry of Health and Medical Education (IMHME). Additionally, the ethics Committee of the Rafsanjan University of Medical Sciences has approved all the protocols of the present study(Ethical codes: IR.RUMS.REC.1400.182), and all the research was performed in accordance with the relevant guidelines/regulations. Participation in the RCS study was voluntary and upon signature of an informed consent form for the interview, physical examinations, the collection of the biological specimens and the use of the collected data for research. The confidentiality of the personal data of the participants was ensured by all necessary measures.

### DATA collection and measurements

Validated comprehensive questionnaires on the demographic features, medical history and habits (e.g., smoking and substance use) were filled in the interview process by trained experts in a computer system connected to a central server. The participants underwent physical examinations by medical doctors, which was followed by a donation of the biological samples, and the measurement of the anthropometric factors all carried out by trained health-care professionals. Blood biochemical factors were measured using a CPALS analyzer (Coultronics, Margency, France) at the central laboratory of RCS center. Comprehensive information was collected regarding substance use and smoking with detailed questionnaires of RCS, including start-age and duration of usage, drug type, and the routes of administration. We defined the regular opiate use as the consumption of opiate for at least one day per week for at least one year continuously (every week of the year).

A significant number of opiate users also smoke cigarettes (estimated correlation rate in our study 48%). In order to avoid the multicollinearity between opiate use and cigarette smoking, we decided to exclude all subjects with passive cigarette smoking (husband’s smoking cigarettes), to assess the effects of opiate only. In the present study, opiates include raw opium (dried latex obtained from the seed capsules of the opium poppy Papaver somniferum), shireh (a refined and concentrated opium extract by boiling and filtering opium)^30,31^, and heroin. We restricted our study to opiate smoking, and the subjects with oral or injectable routes of administration were excluded from our analysis.

Physical activity of adults was measured using a detailed questionnaire recording participants' self-reported daily activities. Based on the daily physical activity, the *metabolic equivalent* of task (MET) score over 24 h was then calculated. The metabolic equivalent of the task (MET-score) is the ratio of a person's working metabolic rate relative to his/her resting metabolic rate^32,33^. Familial history of the hypertension, diabetes, stroke and chronic headache are based on the self-reported information provided by participants in the interview with the comprehensive medical questionnaires used by RCS.

### Outcomes of interest

Our purpose in this study has been to investigate the association of second-hand opiate smoking with stroke and the stroke risk factors and predictive parameters: body mass index (BMI), diabetes, hypertension, high plasma lipids, high fasting blood sugar (FBS), and chronic headache^26,27^.

The criteria for categorizing study subjects for the above-mentioned outcomes were based on the guidelines of the World Health Organization (WHO)^34,35^. BMI > 25 was considered as overweight/obesity, triglyceride (Tg) ≥ 150 mg/dl as high serum Tg, low density lipoprotein (LDL) > 100 mg/dl as high LDL, and total cholesterol > 200 as high cholesterol. Hypertension is defined as hypertension stage II (systolic blood pressure (SBP) > 160 mmHg or diastolic blood pressure (DBP) > 100 mmHg or being under doctor-prescribed treatment with blood pressure lowering medicines). High FBS is defined as FBS > 125, and diabetes as having an FBS > 125 mg/dl or being under the doctor-prescribed treatment with diabetes medicines^34,35^.

### Statistical analyses

Categorical variables were analyzed by the chi-square test or fisher's exact test (in cases where more than 20% of the expected counts were less than 5). T-test was performed for the continuous variables. Quantitative variables were described as either the mean ± standard deviation or the median (interquartile range) as appropriate, and the categorical variables as the frequency and percentage. The assumption of the normality of the distribution of the data was tested using the normal probability plots (skewness and kurtosis index). Among the continuous parameters tested, only MET-score (physical activity) did not show normality. Therefore, it was described with median (interquartile range), and was analyzed by the Mann–Whitney test.

Bivariate and multiple logistic regression analyses were performed to assess the relationship between husbands’ opiate smoking and the interested outcomes in women. For the recognition of the candidate confounding factors, subject matter knowledge and the related epidemiological literature were used first. Next, separate models at the bivariate level were used to estimate the level of association of each potential confounding variable to the dependent outcome. Confounding candidates, which displayed a p-value < 0.2 in these tests^36,37^, as well as variables known as important in the literature were entered into the multiple logistic regression models. Based on these criteria, the following confounding parameters were used in the adjusted logistic regression models to assess each outcome of interest: stroke (age, education years, BMI, hypertension and first degree familial history of stroke^38,39^), FBS (age, education years, BMI, physical activity and the first degree family history of diabetes), diabetes (age, education years, BMI, physical activity and first degree family history of diabetes), hypertension (age, education years, BMI, physical activity and first degree family history (FH1) of hypertension^39,40^), LDL (age, education years, BMI, hypertension, diabetes and first degree family history of hypertension), TG (age, education years, BMI, physical activity and first degree family history of diabetes), cholesterol (age, education years, BMI, hypertension and first degree family history of hypertension^39,41^), chronic headache (age, education years, hypertension and first degree family history of chronic headache^42^).

Statistical analyses were performed in Stata V.14. All p-values are two-sided, and the p-values < 0.05 and the 95% confidence intervals not including 1 are considered as statistically significant. The p-value for the trend (p-trend) was estimated using post-estimation contrast test after logistic regression analysis for the categorized duration of passive opiate exposure in association with each outcome of interest.

## Results

### General characteristics of the study participants

A number of 3261 women were identified in RCS whose husbands had also participated in the study. We then excluded women exposed to passive cigarette smoking after marriage (n = 1624), and women who met other exclusion criteria in this study mentioned above in the methods section (n = 96). The current study population includes 1541 women (35–70 years) whose husbands smoke opiate regularly started after marriage, but reported no cigarette smoking (passive opiate smoking (PO)) (14.5%), compared to women living with men with no regular opiate use or cigarette smoking history after marriage (not exposed to passive opiate (NPO)) (85.5%). Table 1 depicts the descriptive data about women in PO/NPO groups for age, education years, age at marriage, BMI, and the physical activity level (MET = metabolic equivalent of task /24 h). While the BMI (p-value = 0.11) and MET (p-value = 0.14) values are not significantly different between the PO and NPO groups; a significant difference was observed between the mean values of age, age at marriage, and education years in the groups of PO and NPO (p-value < 0.0001) (Table 1). Additionally, a significant association was observed between PO and stroke (p-value < 0.01), hypertension (p-value = 0.01), diabetes (p-value < 0.01), FBS > 125 mg/dl (p-values < 0.001), and LDL > 100 mg/dl (p-value = 0.01). No significant association was observed between PO and overweight/obesity (p-value = 0.28), chol >  = 200 (p-value = 0.10), TG > 150 mg/dl (p-value = 0.15), and chronic headache (p-value = 0.07) (Table 1).

**Table 1:** Baseline characteristics of the study women population categorized by husband opiate smoking.

Number (%)	NPO	PO	Total	p-value
**Stroke**				** < 0.01**^**a**^
No	1289 (99.08)	213 (96.38)	1502 (98.69)	
Yes	12 (0.92)	8 (3.62)	20 (1.31)	
**Overweight/obesity**				0.28
No	764 (58.10)	121 (54.26)	885 (57.54)	
Yes	551 (41.90)	102 (45.74)	653 (42.46)	
**Chol > 200 mg/dl**				0.10
No	709 (53.92)	107 (47.98)	816 (53.06)	
Yes	606 (46.08)	116 (52.02)	722 (46.94)	
**FBS > 125 mg/dl**				** < 0.001**
No	1140 (86.69)	173 (77.58)	1313 (85.37)	
Yes	175(13.31)	50 (22.42)	225 (14.63)	
**Hypertension**				**0.01**
No	1019 (77.49)	156(69.96)	1175 (76.40)	
Yes	296 (22.51)	67 (30.04)	363 (23.60)	
**LDL > 100 mg/dl**				**0.01**
No	544 (41.37)	72 (32.29)	616 (40.05)	
Yes	771 (58.63)	151 (67.71)	922 (59.95)	
**Overweight/obesity (BMI > 25)**				0.32
No	764 (58.10)	121 (54.26)	885 (57.54)	
Yes	551 (41.90)	102 (45.74)	653 (42.46)	
**TG ≥ 150 mg/dl**				0.15
No	757 (57.57)	117 (52.47)	874 (56.83)	
Yes	558 (42.43)	106 (47.53)	664 (43.17)	
**Diabetes**				** < 0.01**
No	1049 (79.77)	160 (71.75)	1209 (78.61)	
Yes	266 (20.23)	63 (28.25)	329 (21.39)	
**Chronic headache**				0.07
No	1200 (92.24)	196 (88.69)	1396 (91.72)	
Yes	101 (7.76)	25 (11.31)	126(8.28)	
**Mean ± SD**				
**Marriage age**	20.73 ± 4.17	18.98 ± 3.88		** < 0.0001**
**Education years**	9.35 ± 4.95	6.86 ± 4.21		** < 0.0001**
**Age**	46.82 ± 8.44	49.44 ± 8.32		** < 0.0001**
**BMI**	29.32 ± 4.81	29.88 ± 4.80		0.11
**Median (interquartile range)**				
**MET**	37.95 (36.18–39.73)	35.75 (35.7–39.55)		0.14^b^

Data are given as mean ± SD, absolute number n (percentage), or median (interquartile range).

p-values for differences between categories were obtained using the t-test or Mann–Whitney test for continuous variables and using the χ^2^ or Fisher Exact Test for categorical variables.

Significant values are in bold.

*BMI* body mass index, *MET* metabolic equivalent of task/24 h.

^a^Fisher's exact test.

^b^Mann–Whitney test.

### Relationship of passive opiate exposure with stroke and stroke risk factors and predictive parameters in women

#### Stroke

Our results displayed a significant unadjusted and adjusted increased odds ratio of stroke in association with PO [adjusted OR: 3.43 (95% CI: 1.33–8.82), p-value: 0.01] (Table 2) . 1 to 10 years of PO was associated with a 188% higher odds ratio of stroke (adjusted OR: 2.88 (95% CI: 0.86–9.62), p-value: 0.09), and above 10 years was associated with a 317% increased odds ratio of stroke (adjusted OR, 4.17 (95% CI: 1.27–13.76), p-value: 0.02) (Table 3).

**Table 2:** Estimated unadjusted and adjusted odds ratios for stroke, stroke risk factors and predictive parameters in women, as predicted by husband opiate smoking.

	Unadjusted model		Adjusted model	
	OR (95% CI)	p-value	OR (95% CI)	p-value
**Husband opiate smoking**				
**Stroke**				
	3.98 (1.61–9.85)	< 0.01	3.43 (1.33–8.82)	**0.01**^**a**^
**Chol > 200 mg/dl**				
	1.23 (0.93–1.64)	0.14	1.08 (0.81–1.46)	0.59^b^
FBS > 125 mg/dl				
	1.95 (1.37–2.76)	** < 0.001**	1.58 (1.08–2.30)	**0.02**^**c**^
**Hypertension**				
	1.39 (1.01–1.91)	** < 0.05**	1.05 (0.73–1.51)	0.80^d^
**LDL > 100 mg/dl**				
	1.42 (1.05–1.91)	** < 0.01**	1.37 (1.01–1.87)	**0.04**^**e**^
**Overweight/obesity (BMI > 25)**				
	1.12 (0.84–1.49)	0.43	0.96 (0.71–1.28)	0.78^f^
**TG ≥ 150 mg/dl**				
	1.22 (0.92–1.62)	0.17	1.06 (0.79–1.43)	0.68^g^
**Total diabetes**				
	1.63 (1.19–2.23)	** < 0.01**	1.26 (0.89–1.79)	0.20^g^
**Chronic headaches**				
	1.49 (0.94–2.37)	0.09	1.37 (0.85–2.20)	0.19^h^

Estimated unadjusted and adjusted odds ratios for stroke, high Chol, FBS, hypertension, LDL, overweight/obesity, high Tg, diabetes and chronic headache in women, as predicted by husband opiate smoking.

Significant values are in bold.

*OR* odds ratio, *CI* confidence interval, *LDL* low density lipoprotein, *Chol* cholesterol, *TG* triglyceride.

^a^Adjusted for age, education years, BMI, stage 2 hypertension, diabetes, MET and family history of stroke.

^b^Adjusted for age, education years, BMI, stage 2 hypertension and family history of hypertension.

^c^Adjusted for age, education years, BMI, MET and family history of diabetes.

^d^Adjusted for age, education years, BMI, MET and family history of hypertension.

^e^Adjusted for age, education years, BMI, stage 2 hypertension, diabetes and family history of hypertension.

^f^Adjusted for age, education years, MET and diabetes.

^g^Adjusted for age, education years, BMI, MET and family history of diabetes.

^h^Adjusted for age, education years, stage 2 hypertension and family history of recurrent chronic headache.

**Table 3:** Estimated unadjusted and adjusted odds ratios for stroke, stroke risk factors in women, as predicted by duration (years) of husband opiate smoking after marriage.

	Unadjusted model		Final adjusted model	
	OR (95% CI)	p-value	OR (95% CI)	p-value
**Husband opiate smoking**				
**Stroke (total n = 1525)**				p-Trend = 0.02
Not passive opiate (n = 1301)	Reference			
1–10 years passive opiate (n = 130)	3.41 (1.08–10.73)	0.03	2.88 (0.86–9.62)	0.09^a^
> 10 years passive opiate (n = 94)	4.77 (1.51–15.10)	< 0.01	4.17 (1.27–13.76)	**0.02**^a^
**Chol > 200 mg/dl (total n = 1541)**				p-Trend = 0.05
Not passive opiate (n = 1315)	Reference			
1–10 years passive opiate (n = 131)	0.93(0.65–1.33)	0.69	0.84(0.58–1.22)	0.37^b^
> 10 years passive opiate (n = 95)	1.83(1.20–2.81)	< 0.01	1.55(1–2.40)	0.05^b^
**FBS > 125 (total n = 1541)**				p-Trend = 0.33
Not passive opiate (n = 1315)	Reference			
1–10 years passive opiate (n = 131)	2.02 (1.31–3.11)	< 0.01	1.82 (1.14–2.92)	0.01^c^
> 10 years passive opiate (n = 95)	1.85 (1.11- 3.08)	0.02	1.31 (0.76 2.26)	0.33^c^
**Hypertension (total n = 1541)**				p-Trend = 0.25
not passive opiate (n = 1315)	Reference			
1–10 years passive opiate (n = 131)	1.11(0.73–1.69)	0.62	0.84(0.52–1.37)	0.49^d^
> 10 years passive opiate (n = 95)	1.83(1.18–2.85)	< 0.01	1.34(0.81–2.22)	0.25^d^
**LDL > 100 mg/dl (total n = 1541)**				p-Trend = 0.01
Not passive opiate (n = 1315)	Reference			
1–10 years passive opiate (n = 131)	1.14 (0.79–1.65)	0.478	1.12 (0.77- 1.64)	0.77^e^
> 10 years passive opiate (n = 95)	1.98 (1.24–3.16)	< 0.01	1.88 (1.15 -3.05)	**0.01**^e^
**Overweight/obesity (BMI > 25)** (**total n = 1541)**				p-Trend = 0.54
Not passive opiate (n = 1315)	Reference			
1–10 years passive opiate (n = 131)	1.17(0.82–1.68)	0.39	1.02(0.71–1.48)	0.90^f^
> 10 years passive opiate (n = 95)	1.05(0.69–1.60)	0.81	0.87(0.57–1.34)	0.54^f^
**TG ≥ 150 mg/dl (total n = 1541)**				p-Trend = 0.97
Not passive opiate (n = 1315)	Reference			
1–10 years passive opiate (n = 131)	1.22(0.85–1.74)	0.28	1.11(0.76–1.61)	0.59^g^
> 10 years passive opiate (n = 95)	1.22(0.80–1.85)	0.35	1.01(0.65–1.55)	0.98^g^
**Total diabetes (total n = 1541)**				p-Trend = 0.64
Not passive opiate (n = 1315)	Reference			
1–10 years passive opiate (n = 131)	1.61(1.08–2.41)	0.02	1.38(0.88–2.15)	0.16^g^
> 10 years passive opiate (n = 95)	1.65(1.04–2.61)	0.03	1.13(0.68–1.86)	0.65^g^
**Chronic headaches (total n = 1525)**				p-Trend = 0.04
Not passive opiate (n = 1301)	Reference			
1–10 years passive opiate (n = 130)	1.10 (0.57–2.10)	0.78	1.02 (0.53–1.97)	0.95^h^
> 10 years passive opiate (n = 94)	2.08 (1.14–3.80)	0.01	1.89 (1.02–3.50)	0.04^h^

Estimated unadjusted and adjusted odds ratios for stroke, high Chol, FBS, hypertension, LDL, overweight/obesity, high Tg, diabetes and chronic headache in women, as predicted by husband opiate smoking.

Significant values are in bold.

*OR* odds ratio, *CI* confidence interval,* LDL* low density lipoprotein, *Chol* cholesterol, *TG* triglyceride.

^a^Adjusted for age, education years, BMI, stage 2 hypertension, diabetes, MET and family history of stroke.

^b^Adjusted for age, education years, BMI, stage 2 hypertension and family history of hypertension.

^c^Adjusted for age, education years, BMI, MET and family history of diabetes.

^d^Adjusted for age, education years, BMI, MET and family history of hypertension.

^e^Adjusted for age, education years, BMI, stage 2 hypertension, diabetes and family history of hypertension.

^f^Adjusted for age, education years, MET and diabetes.

^g^Adjusted for age, education years, BMI, MET and family history of diabetes.

^h^Adjusted for age, education years, stage 2 hypertension and family history of recurrent chronic headache.

Post-estimation contrast test indicated a linear trend of increasing odds ratios of stroke with higher years of passive exposure to opiate (p-trend = 0.02), indicating a dose–response effect.

#### Overweight/obesity

No significant unadjusted or adjusted association was observed between the overweight/obesity (BMI > 25) and PO (adjusted OR: 0.96 (95% CI: 0.71–1.28), p-value: 0.78) (Table 2).

#### Diabetes

Unadjusted regression model displayed a significant 63% increased odds ratio of diabetes associated with PO (unadjusted OR: 1.63 (95% CI: 1.19–2.23), p-value < 0.01). However, adjusting for the confounding factors, the results displayed no significant association between diabetes and PO in the studied population (Adjusted OR: 1.26 (95% CI: 0.89–1.79), p-value: 0.20) (Table 2).

#### Hypertension

According to the unadjusted regression model a significant 39% increased odds ratio of hypertension was observed in association with PO (unadjusted OR: 1.39 (95% CI: 1.01–1.91), p-value: 0.04). This association was abrogated adjusting for the confounding factors [adjusted OR: 1.05 (95% CI: 0.73–1.51), p-value: 0.80)] (Table 2).

#### Fasting blood sugar

Our results display significant unadjusted and adjusted association between the PO and the increased odds ratio of FBS > 125 (Adjusted OR: 1.58 (95% CI: 1.08–2.30), p-value: 0.02) (Table 2). Our results did not confirm a dose-responsive effect for this association (< 10 years adjusted OR: 1.82 (95% CI: 1.14- 2.92), p-value: 0.01; > 10 years adjusted OR: 1.31 (95% CI: 0.76–2.26), p-value: 0.33, p-trend = 0.33) (Table 3).

#### Serum lipid profile (LDL, triglyceride and cholesterol)

In the unadjusted and the adjusted regression models, we found a significant association between PO and a respective 42% and 37% increased odds ratio of LDL > 100 [adjusted OR:1.37 (95% CI: 1.01–1.87), p-value: 0.04] (Table 2).

Additionally, a dose–response effect was observed between the number of years of PO and odds ratio of LDL > 100 mg/dl in women. 1 to 10 years of PO was associated with 12% higher odds ratio of LDL > 100 (adjusted OR, 1.12 (95% CI: 0.77–1.64), p-value: 0.77), and above 10 years of PO was associated with 88% increased odds ratio of high LDL (adjusted OR: 1.88 (95% CI: 1.15–3.05), p-value: 0.01, p-trend = 0.01)(Table 3).

In contrast to LDL, high TG and cholesterol levels did not show a significant association with PO in the unadjusted and adjusted regression analyses [TG adjusted OR: 1.06 (95% CI: 0.79–1.43), p-value: 0.68, chol adjusted OR: 1.08 (95% CI: 0.81–1.46), p-value: 0.59] (Table 3).

#### Chronic headache

We did not observe a significant association between PO and the increased odds ratio of chronic headache, as indicated by the unadjusted and adjusted logistic regression analyses [adjusted OR: 1.37 (95% CI: 0.85–2.20), p-value: 0.19) (Table 2)]. However, assessing a dose-dependent effect, we observed that PO longer than 10 years is significantly associated with 89% higher odds ratio of chronic headache in women, showing a linear dose–response trend (p-trend: 0.04) (Table 3).

## Discussion

For the present study, we used data of the RCS study^28^, as one of the district areas of the PERSIAN cohort^29^. Multiple logistic regression analyses were undertaken in 1541 husband-wife dyads assessing the association of partner’s opiate smoking with women’s risk of stroke, overweight/obesity, diabetes, hyperglycemia, dyslipidemia and chronic headache. We found an increased risk of stroke, high LDL and high FBS in women associated with husband’s opiate smoking. Furthermore, a dose-sensitive connection was found between the odds ratios of stroke, high LDL and chronic headache with the years of passive opiate smoking. Our results point to the necessity of future prospective analyses, which further assess whether passive opiate exposure can be considered as an independent risk factor for stroke.

Opiate consumption has been linked by multiple studies to the increased risk of cerebrovascular diseases and stroke, metabolic dysfunction and all-cause mortality^1–11^. However, studies evaluating the association of the second-hand exposure to opiate with the above-mentioned diseases are scarce. We previously found a higher odds ratio of obesity in male young adults exposed to parental opiate smoking (started after childbirth) in a dose-dependent manner (years of consumption of opiate by parent after childbirth). Therefore, our previous results indicated that the environmental impact of opiate smoking may be sufficient to affect the metabolism in youth^24^. Our present results confirm such indirect metabolic effects, assessing the wives of men smoking opiates regularly. Here we observed a significant dose-sensitive increase in the adjusted odds ratio of high LDL, associated with the partner’s years of opiate smoking after marriage. Consistently, a prior animal study by Najafipour et al., on the second-hand opium exposure found that long term passive opium smoke exposure results in higher cholesterol and LDL-cholesterol levels in rabbits^11^. Additionally, our results displayed an elevated adjusted odds ratio of FBS and stroke associated with husband’s opiate smoking in women. The association with stroke displayed a significant dose-sensitive trend. Albeit, small rate of stroke among women in this study, warrants future larger studies to evaluate this observation.

Several underlying biological mechanisms are suggested for the adverse metabolic and neurologic effects of opiates such as the reduction in the total antioxidant capacity and the elevation in the level of reactive oxygen species (ROS). These effects are mediated by a decrease in the function of enzymatic antioxidants such as superoxide dismutase, catalase, and glutathione peroxidase, modification of the gene expression in the target cells through ROS production^18–21,43–45^, an increase in the inflammatory mediators such as C-reactive protein, interleukin-17, and interleukin-1 receptor^46–49^, an increase in homocysteine^50^, and a decrease in adiponectin^51^. The above-mentioned changes are known mediators of vascular cell injury and atherosclerotic plaque formation, dyslipidemia, insulin resistance^52^, apoptosis of neuron cells and cerebrovascular dysfunction^53–60^.

A strength of the present study is the exclusion of women exposed to the husband’s cigarette smoking. Comprehensive data collection on the history of smoking and illicit drug use is one of the strengths of the Rafsanjan Cohort Study (RCS)^28^ as part of the PERSIAN cohort^29^. The high correlation rate of the concurrent opiate misuse and cigarette smoking often complicates studies sought to determine the harmful effects of each. Here, we performed analysis on women exposed to only second-hand opiate smoking, and excluded all subjects exposed to cigarette smoking to avoid multicollinearity between opiate and cigarette smoking.

Another strength of the current study is the complete physical examination for the anthropometric measurements and the blood biochemical tests for the metabolic parameters by the specialized pathology laboratory in RCS center^28^. In addition, the computer-assisted and server-based method of RCS interviews which were performed by trained experts has provided a higher accuracy of data collection and storage in accordance with the PERSIAN cohort protocols^29^.

The main limitation of this research is that it is a cross-sectional study. This limitation makes it difficult to infer a causal relationship between the passive opiate smoking and stroke and the other analyzed metabolic indices. Follow-up rounds of the RCS study, as well as other prospective cohort studies are warranted to further investigate the findings of the present research.

Our study has another limitation that the information on the substance use and smoking is based on self-report of the participants, which may be associated with underreporting bias in substance use, type of the drug or the administration route. Additionally, misclassification due to self-reporting and recall biases is possible to some extent, which can result in the incidence of bias in estimates and some deviation from reality. To the best of our knowledge a limited number of studies have tested the validity of the self-reported opiate use in Iranian populations. Additionally, these limited studies reported varying levels of validity and sensitivity depending on the sex and age profile of the participants, type of the substance and the socio-cultural and geographical characteristics of the study population^61–64^. In RCS, the validity of the substance use self-reporting is estimated only in RCS youth cohort participants (15–35 years)^65^, and remains to be tested in the adult RCS (present study population). In the city where the current study took place, opiate use is associated with rather low social stigma due to the traditional notion on the medical advantages of opiate use, a fact that is reflected in the rather high rate of self-reporting opiate consumption in RCS population^28^, which makes us more confident in the validity of our data on opiate use. Another study in Iran performed in another geographical region which people also use opiate as a traditional medicine with low social stigma, displayed a high rate of sensitivity of the self-reports on opium use in a Turkmen population^63^.

## Conclusion

In conclusion, the results of the present study indirectly implicate the potential environmental impacts of opiate smoking on family members of the users. Our results warrant future studies that assess whether exposure to second-hand opiate exposure could be taken into consideration as an independent risk factor for stroke and metabolic complications.

## Data Availability

The datasets generated and analyzed during the current study are not publicly available and belong to the PERSIAN Adult Cohort Study of Iran, Rafsanjan University of Medical Sciences cohort center but are available from the corresponding author on reasonable request.

## References

[1] Mousavi-Mirzaei SM (2019). Increasing the risk of stroke by opium addiction. J. Stroke Cerebrovasc. Dis..

[2] Ebrahimi H (2018). Opium addiction and ischemic stroke in Isfahan, Iran: A case-control study. Eur. Neurol..

[3] Iranmanesh F, Syfadini R, Mahalati Y, Gadari F, Dehesh T (2021). Comparison of brain magnetic resonance imaging lesions in opium addict and non-addict patients with thrombotic stroke: A case-control study. Addict. Health.

[4] Moadabi Y, Saberi A, Hoseini S, Karimi A, Yousefzadeh-Chabok S (2019). Cerebral hemodynamic abnormalities of patients with ischemic stroke who are opium addicted: A study by transcranial doppler ultrasonography. Iran. J. Neurol..

[5] Hamziee-Moghadam A, Iranmanesh F, Arabpour-Fathabadi A, Mohammadi F (2018). Cerebrovascular reactivity and carotid intima-media thickness in opium dependents: A case-control study. Addict. Heal..

[6] Hamzei-Moghaddam A, Shafa MA, Khanjani N, Farahat R (2013). Frequency of opium addiction in patients with ischemic stroke and comparing their cerebrovascular doppler ultrasound changes to non-addicts. Addict. Health.

[7] Azod L, Rashidi M, Afkhami-Ardekani M, Kiani G, Khoshkam F (2008). Effect of opium addiction on diabetes. Am. J. Drug Alcohol Abuse.

[8] Karam GA (2004). Effects of opium addiction on some serum factors in addicts with non-insulin-dependent diabetes mellitus. Addict. Biol..

[9] Hosseini SK (2011). Opium consumption and coronary atherosclerosis in diabetic patients: A propensity score-matched study. Planta Med..

[10] Mohammadi A (2009). Effect of opium addiction on lipid profile and atherosclerosis formation in hypercholesterolemic rabbits. Exp. Toxicol. Pathol..

[11] Najafipour H, Beik A (2016). The impact of opium consumption on blood glucose, serum lipids and blood pressure, and related mechanisms. Front. Physiol..

[12] Sanjari Moghaddam H (2021). Cognitive impairment in opium use disorder. Behav. Neurol..

[13] Yeoh SL (2019). Cognitive and motor outcomes of children with prenatal opioid exposure: A systematic review and meta-analysis. JAMA Netw. open.

[14] Ersche KD, Clark L, London M, Robbins TW, Sahakian BJ (2006). Profile of executive and memory function associated with amphetamine and opiate dependence. Neuropsychopharmacology.

[15] Egervari G (2020). Chromatin accessibility mapping of the striatum identifies tyrosine kinase FYN as a therapeutic target for heroin use disorder. Nat. Commun..

[16] De Marinis M, Janiri L, Agnoli A (1991). Headache in the use and withdrawal of opiates and other associated substances of abuse. Headache J. Head Face Pain.

[17] Li L, Yu S (2020). Heroin-induced headache in female heroin addicts. J. Int. Med. Res..

[18] Pereska, Z., Dejanova, B., Bozinovska, C. & Petkovska, L. Prooxidative/antioxidative homeostasis in heroin addiction and detoxification. *Bratisl. Lekárske List. Bratislava Med. J.* (2007). 18225476

[19] Womersley JS, Uys JD (2016). S-Glutathionylation and redox protein signaling in drug addiction. Prog. Mol. Biol. Transl. Sci..

[20] Koch T (2009). μ-Opioid receptor-stimulated synthesis of reactive oxygen species is mediated via phospholipase D2. J. Neurochem..

[21] Gutowicz M, Kaźmierczak B, Barańczyk-Kuźma A (2011). The influence of heroin abuse on glutathione-dependent enzymes in human brain. Drug Alcohol Depend..

[22] Pardo B (2019). Contemporary Asian Drug Policy: Insights and Opportunities for Change.

[23] Strain, E., Saxon, A. J. & Hermann, R. Opioid use disorder: Epidemiology, pharmacology, clinical manifestations, course, screening, assessment, and diagnosis. in *UpToDate, Post, TW, Ed. UpToDate. Waltham, MA *[cited 2018 Apr 1] (2015).

[24] Jalali Z, Bahrampour S, Khalili P, Khademalhosseini M, EsmaeiliNadimi A (2021). Cohort-based analysis of paternal opioid use in relation to offspring’s BMI and plasma lipid profile. Sci. Rep..

[25] Najafipour H, Joukar S, Malekpour-Afshar R, Mirzaeipour F, Nasri HR (2010). Passive opium smoking does not have beneficial effect on plasma lipids and cardiovascular indices in hypercholesterolemic rabbits with ischemic and non-ischemic hearts. J. Ethnopharmacol..

[26] Øie LR, Kurth T, Gulati S, Dodick DW (2020). Migraine and risk of stroke. J. Neurol. Neurosurg. Psychiatry.

[27] Caprio FZ, Sorond FA (2019). Cerebrovascular disease: Primary and secondary stroke prevention. Med. Clin..

[28] Hakimi H (2021). The profile of Rafsanjan cohort study. Eur. J. Epidemiol..

[29] Poustchi H (2018). Prospective epidemiological research studies in Iran (the PERSIAN Cohort Study): Rationale, objectives, and design. Am. J. Epidemiol..

[30] Kapoor L (1995). Opium Poppy: Botany, Chemistry, and Pharmacology.

[31] Shakeri R (2016). Opium use, cigarette smoking, and alcohol consumption in relation to pancreatic cancer. Medicine (Baltimore).

[32] Eghtesad S (2017). The PERSIAN cohort: Providing the evidence needed for healthcare reform. Arch. Iran. Med..

[33] Aguilar-Farias N, Brown WJ, Skinner TL, Peeters GG (2019). Metabolic equivalent values of common daily activities in middle-age and older adults in free-living environments: a pilot study. J. Phys. Act. Health.

[34] Organization, W. H. *Classification of Diabetes Mellitus*. (2019).

[35] Organization, W. H. *Global Status Report on Noncommunicable Diseases 2014*. (World Health Organization, 2014).

[36] Hosmer DW, Lemeshow S, Sturdivant RX (2013). Applied Logistic Regression.

[37] Schwender, H., Hosmer, D.W., Lemeshow, S., & May, S. Applied survival analysis: Regression modeling of time-to-event data. (2012).

[38] Fisher M (2008). Stroke and TIA: Epidemiology, risk factors, and the need for early intervention. Am. J. Manag. Care.

[39] Fletcher GS (2019). Clinical Epidemiology: The Essentials.

[40] Cicero AFG, Ertek S (2011). Hypertension and diabetes incidence: Confounding factors. Hypertens. Res..

[41] Nikparvar M (2021). Dyslipidemia and its associated factors in southern Iranian women, Bandare-Kong Cohort study, a cross-sectional survey. Sci. Rep..

[42] Rohmann JL, Rist PM, Buring JE, Kurth T (2020). Migraine, headache, and mortality in women: A cohort study. J. Headache Pain.

[43] Chavez-Valdez R (2013). Opioids and clonidine modulate cytokine production and opioid receptor expression in neonatal immune cells. J. Perinatol..

[44] Lam C-F (2007). High-dose morphine impairs vascular endothelial function by increased production of superoxide anions. J. Am. Soc. Anesthesiol..

[45] Samarghandian S, Afshari R, Farkhondeh T (2014). Effect of long-term treatment of morphine on enzymes, oxidative stress indices and antioxidant status in male rat liver. Int. J. Clin. Exp. Med..

[46] Ghazavi A, Mosayebi G, Solhi H, Rafiei M, Moazzeni SM (2013). Serum markers of inflammation and oxidative stress in chronic opium (Taryak) smokers. Immunol. Lett..

[47] Ghazavi A, Solhi H, Moazzeni SM, Rafiei M, Mosayebi G (2013). Cytokine profiles in long-term smokers of opium (Taryak). J. Addict. Med..

[48] Newville J, Maxwell JR, Kitase Y, Robinson S, Jantzie LL (2020). Perinatal opioid exposure primes the peripheral immune system toward hyperreactivity. Front. Pediatr..

[49] Nabati S (2013). The plasma levels of the cytokines in opium-addicts and the effects of opium on the cytokines secretion by their lymphocytes. Immunol. Lett..

[50] Masoomi M, Azdaki N, Shahouzehi B (2015). Elevated plasma homocysteine concentration in opium-addicted individuals. Addict. Health.

[51] Shahouzehi B, Shokoohi M, Najafipour H (2013). The effect of opium addiction on serum adiponectin and leptin levels in male subjects: A case control study from kerman coronary artery disease risk factors study (KERCADRS). EXCLI J..

[52] Hasheminasabgorji E, Jha JC (2021). Dyslipidemia, diabetes and atherosclerosis: Role of inflammation and ROS-redox-sensitive factors. Biomedicines.

[53] Behl T (2021). Current trends in neurodegeneration: Cross talks between oxidative stress, cell death, and inflammation. Int. J. Mol. Sci..

[54] Chan K-H (2012). Adiponectin is protective against oxidative stress induced cytotoxicity in amyloid-beta neurotoxicity. PLoS ONE.

[55] Guo J, Zhao X, Li Y, Li G, Liu X (2018). Damage to dopaminergic neurons by oxidative stress in Parkinson’s disease. Int. J. Mol. Med..

[56] Gazaryan, I. G. & Ratan, R. R. Oxidative damage in neurodegeneration and injury. (2017).

[57] Aliev G (2014). Oxidative stress mediated mitochondrial and vascular lesions as markers in the pathogenesis of Alzheimer disease. Curr. Med. Chem..

[58] Song J, Choi S-M, Whitcomb DJ, Kim BC (2017). Adiponectin controls the apoptosis and the expression of tight junction proteins in brain endothelial cells through AdipoR1 under beta amyloid toxicity. Cell Death Dis..

[59] Wu M-H (2019). Adiponectin: A pivotal role in the protection against cerebral ischemic injury. Neuroimmunol. Neuroinflamm..

[60] Wang XX, Zhang B, Xia R, Jia QY (2020). Inflammation, apoptosis and autophagy as critical players in vascular dementia. Eur. Rev. Med. Pharmacol. Sci..

[61] Khajedaluee, M., Rezaee, S. A., Valizadeh, N., Hassannia, T. & Paykani, T. Concordance assessment between self-reports of substance use and urinalysis: A population-based study in Mashhad, Iran. *J. Ethn. Subst. Abuse* 1–15 (2020). 10.1080/15332640.2020.1785362 32677552

[62] Rashidian H (2017). Sensitivity of self-reported opioid use in case-control studies: Healthy individuals versus hospitalized patients. PLoS ONE.

[63] Abnet CC (2004). Reliability and validity of opiate use self-report in a population at high risk for esophageal cancer in Golestan, Iran. Cancer Epidemiol. Prev. Biomark..

[64] Ashrafi S (2018). The validity of self-reported drug use with urine test: Results from the pilot phase of Azar cohort study. Heal. Promot. Perspect..

[65] Khalili P (2021). Validity of self-reported substance use: Research setting versus primary health care setting. Subst. Abuse Treat. Prevent. Policy.

